# Lemierre’s syndrome causing profound thrombocytopenia and respiratory failure: a case report: Lemierre’s syndrome with thrombocytopenia

**DOI:** 10.1186/s12959-021-00306-6

**Published:** 2021-07-31

**Authors:** Samantha Below, Elizabeth Williams

**Affiliations:** Medical College of Wisconsin Affiliated Hospitals, 8701 W Watertown Plank Rd, WI 53226 Wauwatosa, USA

**Keywords:** Thrombosis, Thrombocytopenia, Lemierre’s, Fusobacterium

## Abstract

**Background:**

Lemierre’s syndrome is a rare oropharyngeal infection that can lead to metastatic septic emboli and thrombocytopenia. However, current literature does not report an effect on other cell lines, which we report for the first time in this case.

**Case:**

A previously healthy young African American male presented with profound thrombocytopenia and leukopenia. He was tachypenic on presentation which progressed to respiratory failure requiring intubation. Blood cultures grew *Fusobacterium necrophorum* and imaging revealed internal jugular venous thrombosis*.* He was later diagnosed with Lemierre’s syndrome. The patient was treated with zosyn and fully recovered with no residual deficits in hemoglobin, white cell count, platelet level, or renal function.

**Conclusion:**

Lemierre’s syndrome can cause severe disease in otherwise healthy patients via septicemia and widespread metastatic foci which can cause severe illness. While pulmonary complications are most common, there is little report of effects on other organ systems such as bone marrow and the kidneys. Clinicians should readily evaluate for Lemierre’s syndrome as the complications are severe and can lead to multiorgan failure.

## Background

Lemierre’s syndrome, also termed the forgotten disease due to its rarity, is characterized by oropharyngeal infection, most frequently caused by *Fusobacterium necrophorum*. This leads to internal jugular thrombosis and septic thrombophlebitis resulting in multiple metastatic foci [1, 2]. Lemierre’s syndrome causes thrombocytopenia due to the hemagglutinin produced by the anaerobic *Fusobacterium* bacteria which causes aggregation of platelets. Current literature does not report the extent of thrombocytopenia or an effect of Lemierre’s syndrome on other cell lines. Here we describe a case of Lemierre’s syndrome in a previously healthy young man who presented with profound thrombocytopenia (platelet value < 5000/uL) and leukopenia who subsequently required intubation for respiratory failure.

## Case

**Fig. 1:**
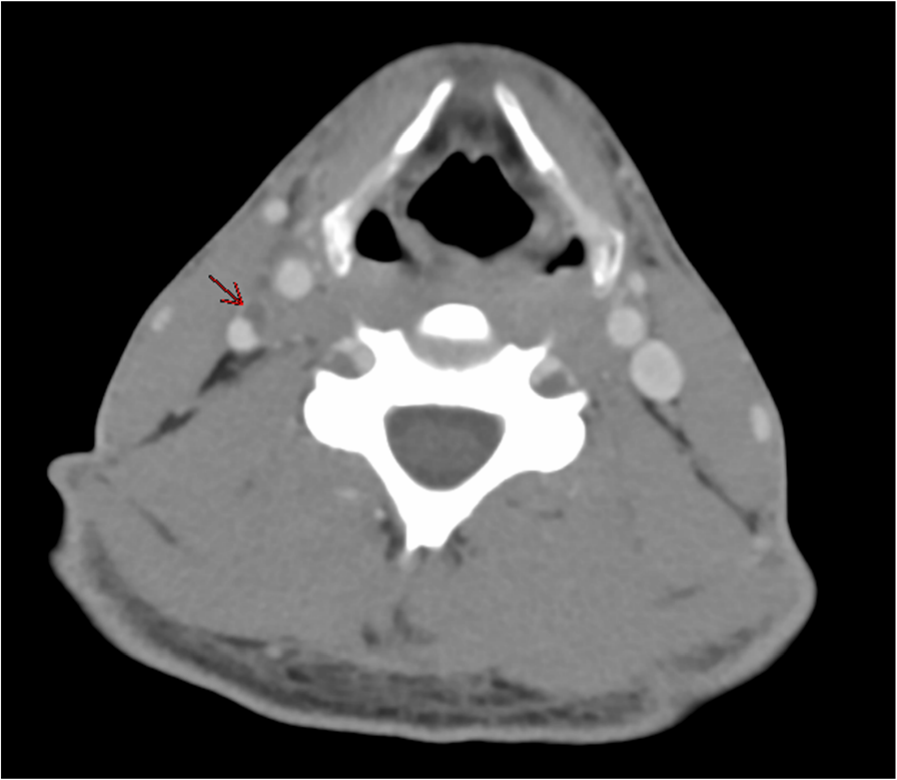
Peripheral filling defect within the mid right internal jugular vein mural enhancement and mild perivascular inflammation

**Fig. 2:**
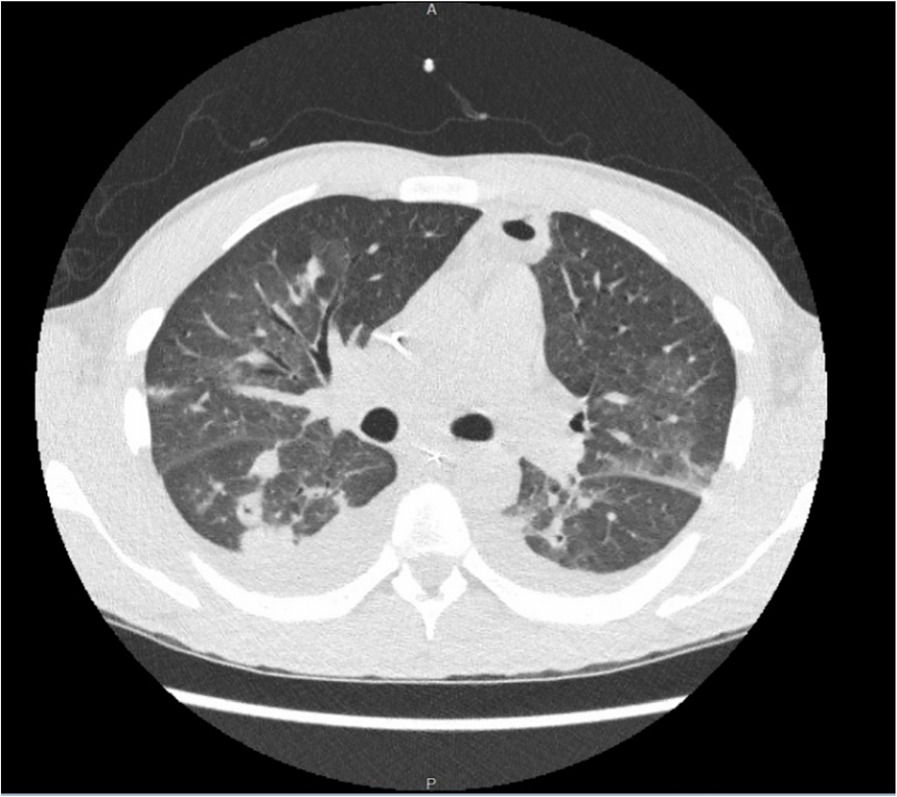
Diffuse interstitial thickening with cavitary changes in multiple bilateral lung nodules with thick walls. Consolidative opacities with some lucent areas which may suggest cavitary changes

A previously healthy 33-year-old African American gentleman presented to the emergency department with a 7-day history of sore throat, nausea, vomiting, and body aches with a 2-day history of dyspnea and hemoptysis. Initial labs revealed severe thrombocytopenia (platelets < 5000/uL), leukopenia (WBC 2.1 × 10^3/uL), and elevated creatinine to 5.16 mg/dL (baseline unknown at presentation). Patient was initially tachycardia and tachypnic ranging from 22 to 35 breaths per minute. A CT chest, abdomen, and pelvis showed bilateral pulmonary parenchymal infiltrates. The patient was started on treatment for presumed community acquired pneumonia. Thrombocytopenia and leukopenia were further evaluated with a bone marrow biopsy and lab studies which were negative for hemolysis or hypoproliferation. Soon after admission, the patient intubated for respiratory distress. Within 24 h of admission, the patient became febrile to 102.9 F and antibiotics were broadened. Blood cultures returned positive for *F. necrophorum* antibiotics were optimized to piperacillin-tazobactam. After 5 days of mechanical ventilation, the patient was extubated. His creatinine improved with fluid administration to 2.35 mg/dL at the time of extubation and a CT neck was revealed an internal jugular vein thrombosis and a CT chest showed cavitary changes consistent with septic emboli (Fig. 1 & Fig. 2). Prior to finishing IV antibiotics, the patient left against medical advice. His creatinine at time of discharge was 0.87 mg/dL his platelets were 331/μL and his white blood cell count improved to 5.3 K/uL (Table 1). Further, as the patient improved from a laboratory standpoint, he did not receive anticoagulation as the cause of the thrombosis was secondary to the infection. The patient has not followed up with the health system to date.

**Table 1:** Unique characteristics of lab trend of patient

Lab component	Value at Admission	Value at Discharge
**White blood cell count (10^3/**μL**)**	2.1	5.3
**Platelets (10^3/μL)**	< 5	331
**Hemoglobin (g/dL)**	12.0	11.7
**Creatinine (mg/dL)**	5.26	0.80

## Discussion

Lemierre’s syndrome is characterized by a bacterial oropharyngeal infection, most frequently with *Fusobacterium necrophorum*, and internal jugular vein septic thrombophlebitis leading to septicemia with multiple metastatic foci [1–3]. This typically presents in young patients with a median age of 21 (Q1-Q3: 17–33) with no prior comorbidities [2]. Making the severity of illness alarming in these young patients.

Metastatic foci in Lemierre’s syndrome cause deleterious complications in other organs. Emboli most frequently spread to the lung, with reports of 71% of patients [2–4]. It has been reported that the most common site of metastasis with Lemierre’s syndrome is the lungs, in fact pulmonary emboli can occur as early as the first day of septicemia [5]. Less commonly, complications such as ARDS, as in our patient, may arise [4]. The cause of our patient’s kidney failure may be secondary poor perfusion of the kidney as the renal in nature as it improved with fluid administration.

In Lemierre’s syndrome, patients’ lab work frequently reveals thrombocytopenia, most likely due to the production of hemagglutinin and lipopolysaccharides by *F. necrophorum* [2, 5, 6]. The lipopolysaccharides content of *F. necrophorum* is higher than other gram-negative bacteria which is the main attribute to why thrombocytopenia occurs in patients with a *F. necrophorum* infection [6]. To our knowledge, no reports exist in the literature of patients with Lemierre’s syndrome presenting with leukopenia and the degree of thrombocytopenia seen in our case. However, it may be more likely that the leukopenia was caused by a hemagglutination process like that which causes thrombocytopenia. To our knowledge this is the first case of Lemierre’s syndrome where multiple lines decreased.

## Conclusion

Lemierre’s syndrome should be considered in patients presenting with severe thrombocytopenia and history of pharyngeal infection. While multiple parameters were affected in our patient, white blood cell count, platelets, and creatinine, with antibiotic treatment, our patient completely recovered what could have been a fatal disease. As in our case, patients can rarely present with severe respiratory distress requiring intubation. Lemierre’s syndrome should not be overlooked as it is curable with antibiotics. Lemierre’s syndrome should consistently be in the differential diagnosis for sepsis in previously healthy individuals with new onset thrombocytopenia with or without leukopenia. Further, there may be additional effects of Lemierre’s syndrome on the renal system and other blood lines that are not recognized routinely.

## Data Availability

Supporting data is available.

## Abbreviations

ARDS: Acute respiratory distress syndrome
HUS: Hemolytic uremic syndrome
TTP: Thrombotic thrombocytopenic purpura
WBC: White Blood count
